# No impact of disease duration on response to tildrakizumab treatment among patients with moderate‐to‐severe plaque psoriasis: Post hoc analyses from two phase 3 (reSURFACE 1 and reSURFACE 2) and one phase 4 (TRIBUTE) studies

**DOI:** 10.1002/ski2.263

**Published:** 2023-06-28

**Authors:** Johannes Griss, Gudrun Ratzinger, Julia‐Tatjana Maul, Wolfgang Weger, Diamant Thaçi, José Manuel Carrascosa, Constanze Jonak

**Affiliations:** ^1^ Department of Dermatology Medical University of Vienna Vienna Austria; ^2^ Department of Dermatology Medical University of Innsbruck Innsbruck Austria; ^3^ Department of Dermatology University Hospital Zurich Zurich Switzerland; ^4^ Faculty of Medicine University of Zurich Zurich Switzerland; ^5^ Department of Dermatology and Venereology Medical University of Graz Graz Austria; ^6^ Institute and Comprehensive Centre for Inflammation Medicine University of Lübeck Lübeck Germany; ^7^ Department of Dermatology Hospital Universitari Germans Trias i Pujol, IGTP, UAB Badalona Spain

## Abstract

In the literature there is no consensus on the correlation between early systemic intervention and better treatment response in psoriasis. Here we present data on the impact of disease duration (<5 years, 5‐<10 years, and ≥10 years) on response to tildrakizumab treatment among patients with moderate‐to‐severe plaque psoriasis from the reSURFACE 1 and reSURFACE 2 phase 3 trials and the TRIBUTE phase 4 study. Overall, there was no significant effect of disease duration on the Psoriasis Area and Severity Index ≤1, ≤3, and ≤5, or the Dermatology Life Quality Index 0‐1 response rates. Tildrakizumab was highly effective regardless of the psoriasis disease duration.

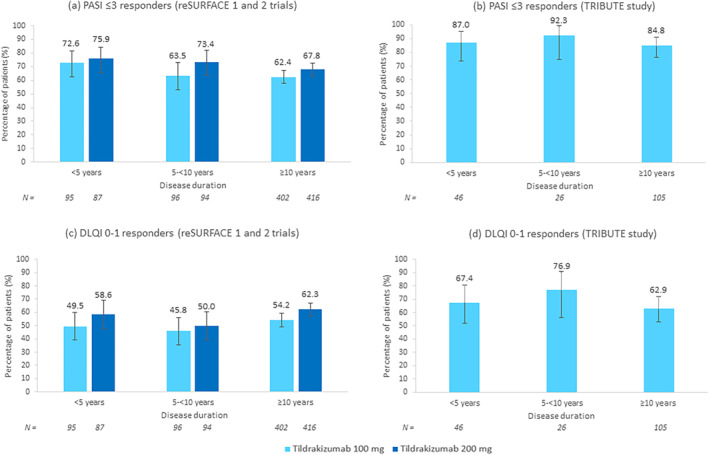

Dear Editor

Psoriasis is a chronic inflammatory skin disease associated with a number of comorbidities. Undertreated psoriasis can result in prolonged subclinical inflammation significantly increasing patients' risks for developing comorbidities, which in turn may not only complicate the management of the disease but also impact on patients' health‐related quality of life. There is no clear consensus in the literature regarding a direct correlation between early systemic intervention and better treatment response in psoriasis. However, an early intervention within 2 years of disease‐onset may lead to improved clinical response.^1^ Tildrakizumab is a monoclonal antibody targeting interleukin‐23p19 approved for the treatment of moderate‐to‐severe plaque psoriasis that has demonstrated a sustained long‐term disease control, with a favourable safety profile, both in randomized clinical trials and in the real‐world setting.^2^, ^3^, ^4^ The aim of this analysis was to describe the impact of disease duration on response to tildrakizumab treatment among patients with moderate‐to‐severe psoriasis from the phase 3 reSURFACE 1 and 2 and the phase 4 TRIBUTE studies.^2^, ^5^

Both reSURFACE 1 (64‐week; NCT01722331) and reSURFACE 2 (52‐week; NCT01729754) were double‐blinded, randomized controlled trials, while the TRIBUTE (24‐week; EudraCT 2019‐002804‐42) was an open‐label trial that resembled real‐life clinical practice.^2^, ^5^ Adult patients with moderate‐to‐severe plaque psoriasis were included. In the reSURFACE trials, tildrakizumab 100 and 200 mg were administered at week 0, week 4, and every 12 weeks thereafter. Pooled results of patients who continued treatment with the same tildrakizumab dose up to week 28 (*N* = 1190) are presented.^2^ In the TRIBUTE study, tildrakizumab 100 mg was administrated according to the Summary of Product Characteristics and analyses were conducted using 24‐week data from the safety population (*N* = 177). Disease duration was divided into 3 categories as follows: <5 years, 5‐<10 years, and ≥10 years. Response to treatment was assessed based on the proportions of patients achieving absolute Psoriasis Area and Severity Index (PASI) ≤1, ≤3 or ≤5 (PASI ≤1/≤3/≤5 responses), or a Dermatology Life Quality Index (DLQI) of 0 or 1 (DLQI 0–1 response) by disease duration groups. PASI ≤3 response was also assessed by sex and body weight (≤90/>90 kg) within each disease duration group. Missing data were imputed using the Last Observation Carried Forward method.

Baseline characteristics were similar among disease duration groups and were also similar to those of the overall trial populations.^2^, ^5^ The proportions of patients treated with tildrakizumab stratified by disease duration who achieved absolute PASI scores ≤3 at week 28 (reSURFACE 1 and 2) or at week 24 (TRIBUTE) are shown in Figure 1a,b, respectively. Although PASI ≤3 responses were similar across disease duration groups, a numerically higher proportion of patients achieving a PASI ≤3 response was observed among patients with a shorter disease duration. Comparable results were found for PASI ≤1/≤5 responses (data not shown). Similarly, the proportions of tildrakizumab patients stratified by disease duration who achieved DLQI 0–1 scores at week 28 (reSURFACE 1 and 2) or at week 24 (TRIBUTE) are shown in Figures 1c,d, respectively. DLQI 0–1 responses were also similar across disease duration groups. Analyses performed by sex and body weight across disease duration groups showed comparable PASI ≤3 responses within and between disease duration groups (data not shown).

**FIGURE 1:**
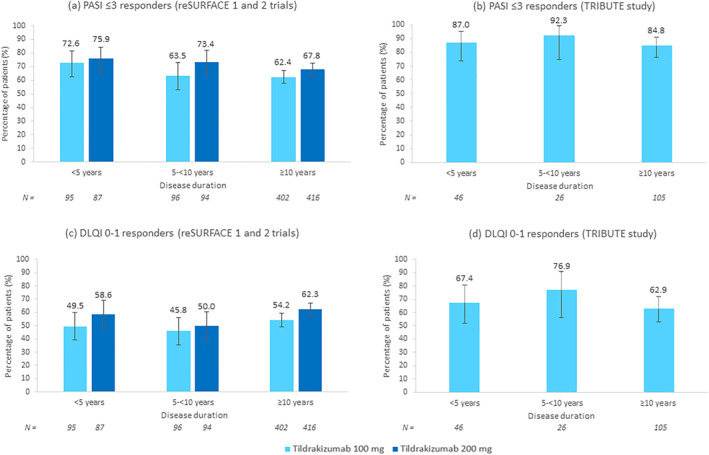
PASI ≤3 and DLQI 0–1 response rates at week 28 (reSURFACE 1 and 2 trials) and at week 24 (TRIBUTE study) by disease duration. (a) PASI ≤3 responders to tildrakizumab 100 and 200 mg in the reSURFACE 1 and 2 trials. (b) PASI ≤3 responders to tildrakizumab 100 mg in the TRIBUTE study. (c) DLQI 0–1 responders to tildrakizumab 100 and 200 mg in the reSURFACE 1 and 2 trials. (d) DLQI 0–1 responders to tildrakizumab 100 mg in the TRIBUTE study. Error bars represent 95% confidence intervals. DLQI, Dermatology Life Quality Index; PASI, Psoriasis Area and Severity Index.

Overall, there was no significant effect of disease duration on the response rates to tildrakizumab. Regardless of gender or body weight, tildrakizumab showed high and comparable levels of efficacy across disease duration groups. Of note, a high percentage of patients (84.8%) with a long disease duration (≥10 years) achieved a PASI ≤3 after 24 weeks of tildrakizumab treatment in the real‐world setting. Our results are in line with published data on other interleukin‐23p19 inhibitors such as guselkumab showing that duration of psoriasis (<15 vs. ≥15 years) was not inversely associated with PASI 90 response.^6^, ^7^ However, our analysis suggests a trend towards a better PASI ≤3 response among patients with a disease duration <10 years. Similar findings were previously observed in a post hoc analysis of the reSURFACE trials reporting a decrease in mean duration of psoriasis as relative PASI response at week 28 improved.^8^ An important limitation of these analyses is the small number of patients with 5‐<10 years of disease duration in the TRIBUTE study. However, the reSURFACE trials included a large number of patients in all disease duration categories.

In conclusion, tildrakizumab is highly effective regardless of psoriasis disease duration.

## CONFLICT OF INTEREST STATEMENT

Johannes Griss reports honoraria for training sessions or participation in advisory boards from Abbvie, Novartis, and Sandoz. Gudrun Ratzinger reports honoraria for training sessions or participation in advisory boards from Abbvie, Almirall, Eli Lilly, Janssen, Leo Pharma, Novartis, Pelpharma, and UCB. Julia‐Tatjana Maul has served as advisor and/or received speaking fees and/or participated in clinical trials sponsored by AbbVie, Almirall, Amgen, Bristol‐Myers Squibb, Celgene, Eli Lilly, Janssen, Leo Pharma, Merck Sharp & Dohme, Novartis, Pfizer, Pierre Fabre, Roche, Sanofi, and UCB. Wolfgang Weger has received speaker and/or consulting honoraria and/or travel refunds from AbbVie, Almirall, Amgen, Celgene, Eli Lilly, Janssen, Leo Pharma, Merck Sharp & Dohme, Novartis, Pfizer, and Sandoz. Diamant Thaçi has received honoraria as an advisor, speaker and/or investigator from AbbVie, Almirall, Amgen, Biogen‐Idec, Boehringer Ingelheim, Bristol‐Myers Squibb, Eli Lilly, Galapagos, Galderma, Janssen, Leo Pharma, Novartis, Pfizer, Regeneron, Roche‐Possay, Samsung, Sanofi, and UCB. José Manuel Carrascosa has participated as principal investigator/site investigator, and/or member of steering committees and/or advisor and/or invited speaker for AbbVie, Almirall, Amgen, Celgene, Eli Lilly, Janssen, Leo Pharma, Mylan, Novartis, and Sandoz. Constanze Jonak served as principal investigator (grant paid to institution), and/or received speaker and/or consulting honoraria, and/or travel refunds from AbbVie, Almirall, Amgen, Bristol‐Myers Squibb, Boehringer Ingelheim, Eli Lilly, Innate, Janssen, Kyowa Kirin, Leo Pharma, Novartis, Pfizer, Recordati, Sandoz, Takeda, UCB, and 4SC.

## AUTHOR CONTRIBUTIONS

**Johannes Griss**: Conceptualization (equal); Writing – review & editing (equal). **Gudrun Ratzinger**: Conceptualization (equal); Writing – review & editing (equal). **Julia‐Tatjana Maul**: Conceptualization (equal); Writing – review & editing (equal). **Wolfgang Weger**: Conceptualization (equal); Writing – review & editing (equal). **Diamant Thaçi**: Conceptualization (equal); Writing – review & editing (equal). **José Manuel Carrascosa**: Conceptualization (equal); Writing – review & editing (equal). **Constanze Jonak**: Conceptualization (equal); Writing – review & editing (equal).

## FUNDING INFORMATION

Almirall R&D, Barcelona, Spain.

## ETHICS STATEMENT

The original studies were conducted following the Declaration of Helsinki principles and were approved by the ethics committee at each site.

## Data Availability

The data that support the findings of this study are available from the corresponding author upon reasonable request.
